# Ontogenetic foraging activity and feeding selectivity of the Brazilian endemic parrotfish *Scarus zelindae*

**DOI:** 10.7717/peerj.2536

**Published:** 2016-10-12

**Authors:** Pedro H.C. Pereira, Marcus Santos, Daniel L. Lippi, Pedro Silva

**Affiliations:** 1 Department of Marine Biology, James Cook University, Townsville, Queensland, Australia; 2 Coral Reef Ecosystem Department, Reef Conservation Project, Recife, PE, Brazil; 3 Department of Oceanography, Universidade Federal de Pernambuco, Recife, PE, Brazil; 4 Biological Sciences Department, Federal Institute of Education, Science and Technology (IFGoiano), Rio Verde, GO, Brazil

**Keywords:** Feeding behaviour, Resource availability, Parrotfishes, Southwestern Atlantic Ocean, Brazilian reefs

## Abstract

Parrotfish are fundamental species in controlling algal phase-shifts and ensuring the resilience of coral reefs. Nevertheless, little is known on their ecological role in the south-western Atlantic Ocean. The present study analysed the ontogenetic foraging activity and feeding selectivity of the Brazilian endemic parrotfish *Scarus zelindae* using behavioural observation and benthic composition analyses. We found a significant negative relationship between fish size and feeding rates for *S. zelindae* individuals. Thus, terminal phase individuals forage with lower feeding rates compared to juveniles and initial phase individuals. The highest relative foraging frequency of *S. zelindae* was on epilithic algae matrix (EAM) with similar values for juveniles (86.6%), initial phase (88.1%) and terminal phase (88.6%) individuals. The second preferred benthos for juveniles was sponge (11.6%) compared with initial (4.5%) and terminal life phases (1.3%). Different life phases of *S. zelindae* foraged on different benthos according to their availability. Based on Ivlev’s electivity index, juveniles selected EAM and sponge, while initial phase and terminal phase individuals only selected EAM. Our findings demonstrate that the foraging frequency of the endemic parrotfish *S. zelindae* is reduced according to body size and that there is a slight ontogenetic change in feeding selectivity. Therefore, ecological knowledge of ontogenetic variations on resource use is critical for the remaining parrotfish populations which have been dramatically reduced in the Southwestern Atlantic Ocean.

## Introduction

Species may select vital resources (e.g., habitat, food and mates) according to their availability in natural communities. Yet, these resources are subjected to temporal and spatial fluctuation that influences species patterns of resource use (Holling, 1973; Holt, Rutherford & Peterman, 2008; Pekkonen, Ketola & Laakso, 2013). Many coral reef fishes change their distribution and diet according to the availability of food resources; a trend that has already been investigated for many families such as Haemulidae (Pereira & Ferreira, 2013), Pomacentridae (Frédérich et al., 2009; Waldner & Robertson, 1980) and Scaridae (Plass-Johnson, McQuaid & Hill, 2013). For instance, parrotfish behavior seems to change in response to food resource availability, meaning local variation in algal abundance can influence fish feeding preferences and modify parrotfish patterns of abundance (Russ, 2003; Hoey, Pratchett & Cvitanovic, 2011).

Parrotfishes are believed to be important contributors to healthy reefs because they consume algae that compete with corals for space in tropical waters (Hughes et al., 2003; Graham et al., 2013). Grazing activity also provides open space for coral recruitment, securing better conditions for coral reef development during recent strong impacts such as climate change and global warming (Bennett et al., 2015). As a generalist group, parrotfish foraging activity varies strongly according to morphology, life phase, and food availability (Bonaldo, Hoey & Bellwood, 2014). They are usually classified in three main functional groups: browsers, scrapers and excavators (Bellwood & Choat, 1990; Streelman et al., 2002; Francini-Filho et al., 2008; Bonaldo, Hoey & Bellwood, 2014). *Browsers* tend to cut off macroalgae, leaving no scars on the substrate (e.g., *Sparisoma* spp.), *scrapers* feed at high rates leaving only a superficial scrape and normally do not damage coral surface (e.g., *Scarus* spp.) and *excavators* feed at low rates removing large portions of the substratum or coral using their robust jaws, leaving noticeable scars (e.g., *Bolbometopon muricatum*) (Bellwood & Choat, 1990; Streelman et al., 2002).

Ontogenetic changes in foraging activity and feeding preference are relevant for many coral reef fishes, including parrotfish (Bellwood, 1988; Pereira & Ferreira, 2013). Bellwood, Hughes & Hoey (2006) suggested that newly settled *Scarus* individuals feed on crustaceans, whilst larger juveniles almost exclusively ingest algae and detritus. Additionally, morphological and anatomical body changes throughout ontogeny also directly influence parrotfish feeding preferences. As parrotfish grow, the enlargement and development of the oral jaws and associated musculature allow them to bite deeper into the benthos, effectively scraping or even excavating the substratum (Bellwood & Choat, 1990; Bonaldo, Hoey & Bellwood, 2014; Francini-Filho et al., 2008). Although much research has been conducted analysing ontogenetic changes on parrotfish ecology in the Indo-Pacific and Caribbean, few studies have attempted to analyse variations on foraging activity and feeding preference across different life stages in endemic parrotfish species of the Southwestern Atlantic Ocean.

*Scarus zelindae* is an endemic parrotfish from Brazilian waters occurring on coral and rocky reefs at depths up to 60 m. Previous studies have shown that *S. zelindae* is predominantly herbivorous, ingesting algae and detritus (Ferreira & Gonçalves, 2006). Francini-Filho et al. (2010) found *S. zelindae* had a preference for turf algae and classified this species as a scraper. However, larger terminal phase individuals can also act as excavators (Francini-Filho et al., 2008; Francini-Filho et al., 2010), whereas juveniles have been recorded feeding on *Millepora* spp. fire-corals with feeding rates of up to 0.58 ± 0.35 bites/min (Pereira et al., 2012). Nevertheless, these preliminary studies were more naturalist and did not systematically test for ontogenetic changes on *S. zelindae* resource use. Therefore, the relationship of their ontogenetic foraging activity and feeding selectivity is still unclear. The ecological role of parrotfish on tropical coral reefs is evident; hence, it is critical to better understand ontogenetic changes in their feeding patterns and the different effects parrotfish have on benthic communities according to size. Adults are normally targeted by local fisheries and the large bodied individuals could be the most effective individuals controlling algal growth. However, this has never been analysed for *Scarus* individuals in the Southwestern Atlantic Ocean. If *S. zelindae* display ontogenetic changes in feeding activity and foraging preferences, then individuals of different life phases could have a disproportional ecological role in shaping benthic communities.

The present study aims to understand the ontogenetic foraging activity and feeding selectivity of the endemic parrotfish *S. zelindae* on tropical coral reefs. To achieve this goal, the foraging intensity and feeding behaviour of juvenile, initial phase (IP) and terminal life phase (TP) individuals were recorded using behavioural observations. The benthic composition at foraging sites was also examined to determine resource availability relative to foraging behavior. Specifically, we analysed if individuals of different life phases selected food resources according to substratum availability or whether they showed preferences for particular food types. 

## Methods

### Study area

The studied coral reef complex is located within the limits of the “Costa dos Corais” marine protected area (MPA) which encompasses 135 km of coastline in Pernambuco State of northeastern Brazil. The “Costa dos Corais” MPA was the first Brazilian federal conservation area that included coastal reefs and is the largest multiple-use MPA in the country (Maida, & Ferreira, 1997). Deeper reefs (from 25 to 35 meters depth) (8°49^′^S and 35°03^′^W) were used as sampling sites, which comprised a series of continuous reef tracts with sand intervals and high structural complexity. The benthic community is mainly composed of epilithic algae matrix, coralline algae, sponges and hard corals Video S1). Deeper reefs were used as sampling sites considering that the shallow reefs have been extremely impacted by spearfishing and it is currently difficult to observe *S. zelindae* terminal phase individuals in these areas (PHC Pereira, pers. comm., 2016). Therefore, these deeper reefs represent a unique opportunity to analyse parrotfish ontogenetic foraging activity and feeding selectivity because all the different life phases have a representative abundance for behavioural observations.

### Foraging activity

**Figure 1:**
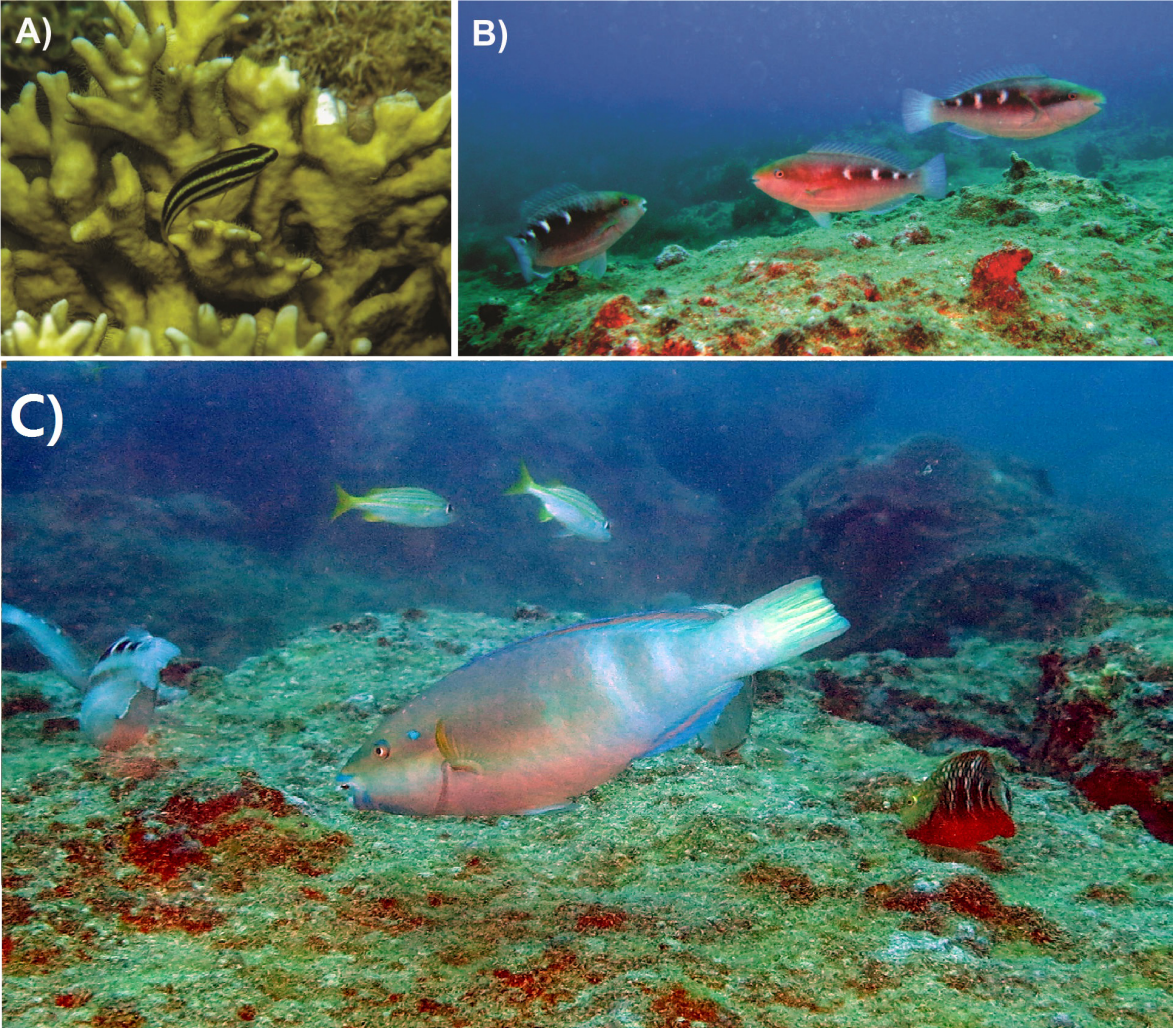
*Scarus zelindae* life phase classification highlighting different color pattern. (A) Juvenile; (B) Initial Phase (IP) and (C) Terminal Phase (TP). Photographs by PHC Pereira.

Feeding rates (bites per minute) of *S. zelindae* individuals were obtained from animal focal sampling that was always carried out by one observer (Altmann, 1974). Dives were conducted by SCUBA from December 2014 to March 2015. Individuals were observed over 5 min intervals, except when the individuals evaded the observer. On average, a minimum distance of 5 meters was maintained between the observer and each fish in order to reduce observer impact on fish behaviour (Pereira, Leal & Araújo, 2016) whilst increasing identification accuracy of feeding selectivity. During each observation session divers recorded feeding rates (total number of bites) of each individual and the substratum type where feeding was observed. Fish size (total length - TL) was visually estimated and individuals were categorized as juvenile, initial and terminal phase according to size. Individuals were also classified into different life phases based upon variation in their patterns of coloration (Fig. 1). A total of 20 individuals from each life phase (juvenile, initial and terminal phase) were recorded during 5 min observation sessions totalling 300 min of direct observation.

### Benthic community

The benthic composition was also analyzed in the reef complex where the foraging behaviour of *S. zelindae* was recorded, using the point intercept transect method (Meese & Tomich, 1992). A 20 meter transect belt was used in which the diver registered the substrate at 0.5 m intervals. In order to avoid temporal variations in resource availability all the benthic surveys were performed during the same dives and same period as feeding behavioral observations (from December 2014 to March 2015). A total of 20 randomly distributed belt transects were conducted along the top of the reef at an average depth of 25 m. The benthic community was classified using the categories: epilithic algal matrix (EAM), coralline algae, sand, sponge, hard coral, macroalgae and bare rock.

### Data analyses

One-way analysis of variance (ANOVA) was used to compare mean feeding rates of *S. zelindae* individuals at different life phases. Post hoc comparisons based on Tukey HSD test were subsequently made for the significant factors using Statistica 10 (StatSoft Inc. Tulsa, OK, USA). Linear regressions were used to compare the bite rates (bites/min^−1^) with parrotfish body size (cm).

To test differences between the relative foraging frequency of *S. zelindae* individuals on different benthic categories we applied a permutational multivariate analysis of variance (PERMANOVA). *S. zelindae* foraging frequency data on different benthic categories were log transformed (*X* + 1) and reassembled in a Bray-Curtis similarity matrix. Unrestricted permutation of raw data was used as the best technique for analyzing one factor. A permutational analysis of multivariate dispersions (PERMIDISP) was also applied to analyze whether the multivariate variations were homogeneous or not (Anderson, 2001; Anderson, & Walsh, 2013). PERMANOVA and PERMIDISP were conducted using Primer-e 6 PERMANOVA+1.0 software (Ver. 6.1.14) 227 (Anderson & Gorley, 2007).

Principal component analysis (PCA) was used to investigate correlations between *S. zelindae* feeding preferences and individual life phases, with the total number of bites per substratum category used as the main data. All the data were standardized and log-transformed prior to multivariate analyses. PCA was performed using Primer-e 6 PERMANOVA+1.0 software (Ver. 6.1.14).

Ivlev’s electivity index (Ivlev, 1961) was calculated to examine the ontogenetic feeding selectivity of *S. zelindae* individuals. The index was calculated using the following equation:

\begin{eqnarray*}{E}_{i}= \frac{{r}_{i}-{p}_{i}}{{r}_{i}+{p}_{i}} \end{eqnarray*}

where electivity (*E*_*i*_) for each benthic category *i* was calculated from the proportional availability of that benthic category (*p*_*i*_) in the field and the proportional of feeding bites on that benthic category (*r*_*i*_). The values of *E* can vary from −1.0 to +1.0 with negative values indicating avoidance, zero indicating random selection, and positive values indicating active selection. In order to estimate 95% confidence intervals of Ivlev’s index values, bootstrapping procedures (9,999 simulations) were performed on individual feeding rates (keeping resource availability constant). Variability analyses were performed following procedures used by Smith (1982).

## Results

### Feeding activity

The feeding rate of *S. zelindae* was 34.6 ± 6.6 bites/min^−1^ (mean ± s.d.) for juveniles, 17.9 ± 4.9 for initial phase and 14.9 ± 4.6 for terminal phase individuals. Significant differences in foraging rates were observed among life phases (ANOVA; *F* = 224.56; *p* < 0.01). Tukey HSD test showed significant differences between juveniles and initial phase (*p* < 0.01) and also between juveniles and terminal phase (*p* < 0.01). However, no significant difference in foraging rate was observed between initial phase and terminal phase (*p* = 0.10).

**Figure 2:**
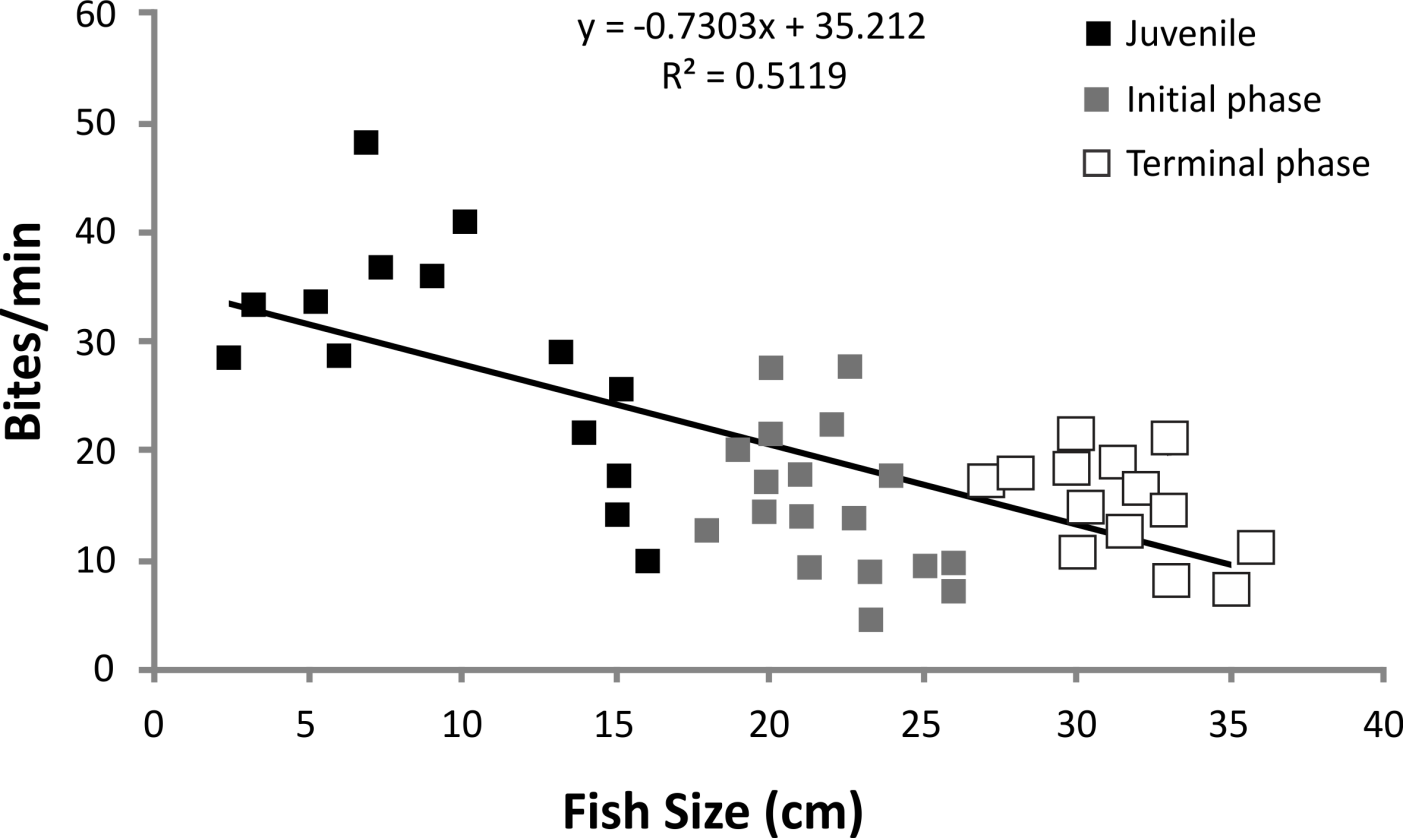
Linear regression of *S. zelindae* feeding rates (bites/min^−1^) compared with fish size (cm). Each point represents an individual. Size of *S. zelindae* ranged from 2.5 cm to 36 cm.

There was a significant negative relationship between fish size (cm) and feeding rates (bites/min^−1^) for *S. zelindae* individuals (*R*^2^ = 0.51; *p* = 0.008) indicating a reduction in feeding rate with increasing fish size (Fig. 2).

**Figure 3:**
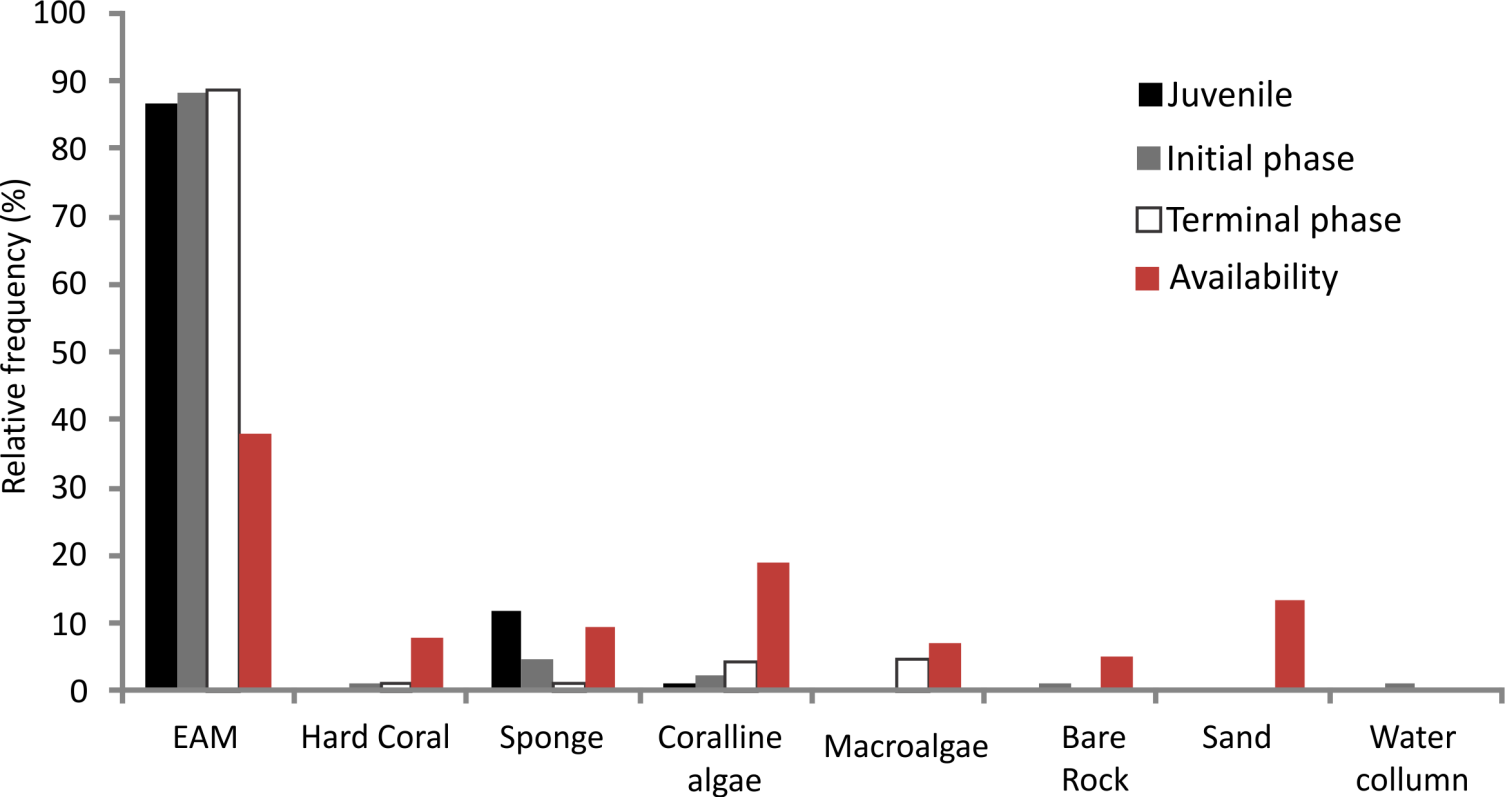
Relative frequency in foraging of *S. zelindae* individuals on different substratum per life phase and relative abundance of the benthic composition (resource availability).

The relative foraging frequency of *S. zelindae* was highest on the EMA and this was similar for all life phases; juveniles (86.6%), initial phase (88.1%) and terminal phase (88.6%) (Fig. 3). On the other hand, sponge was the second highest preferred feeding substratum for juveniles (11.6%) foraged at a higher percentage compared with other life phases: initial phase (4.5%) and terminal phase (1.3%). Terminal phase individuals displayed a considerable foraging frequency on coralline algae (4.3%) and macroalgae (4.5%) (Fig. 3). No significant difference was recorded for the relative frequency of foraging comparing *S. zelindae* individuals at different life phases (PERMANOVA; Pseudo *F* = 1.31; *p* = 0.21).

**Figure 4:**
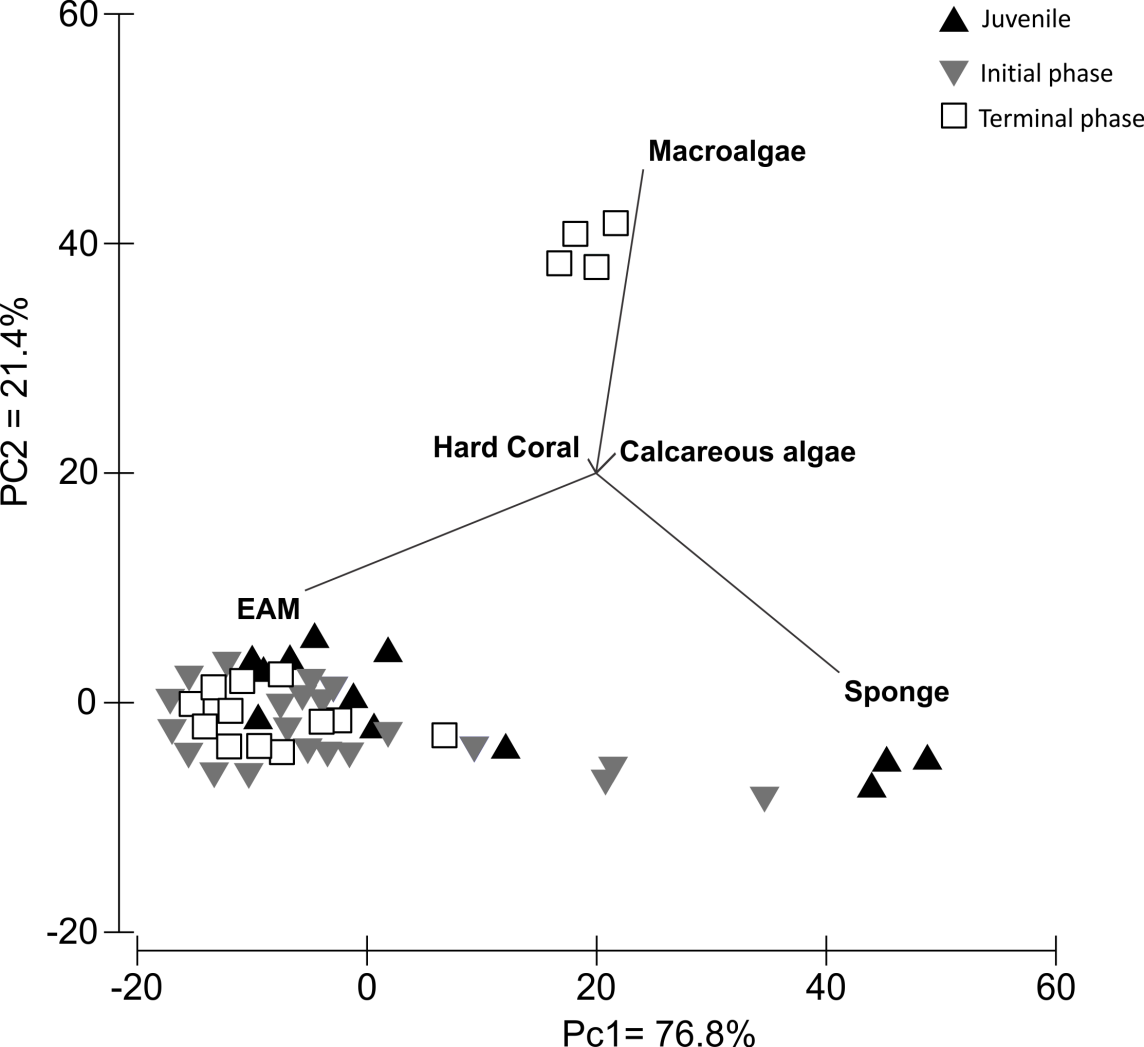
Principal components analysis with data clustered by types of substrata used as a food resource for *S. zelindae* at different life phases.

The PCA analysis of *S. zelindae* foraging preference explained 98.1% of the total variability; 76.8% PC1 and 21.4% PC2, respectively. The eigenvalue for PC1 was 262 and 73 for PC2. The analysis confirmed that EAM was the most used food resource for all life phases (Fig. 4). However, for juvenile individuals sponge was the second most foraged resource, whereas, in terminal phase individuals it was macroalgae (Fig. 4).

### Substratum availability

The benthic substratum at feeding sites of *S. zelindae* was mainly composed of EAM (38.0%), coralline algae (18.7%) and sand (13.5%), which together represented more than 70% of the benthic composition. The less representative categories were rock (5.2%) and macroalgae (7.2%) which represented less than 15% of the benthos (Fig. 3).

### Foraging selectivity

**Figure 5:**
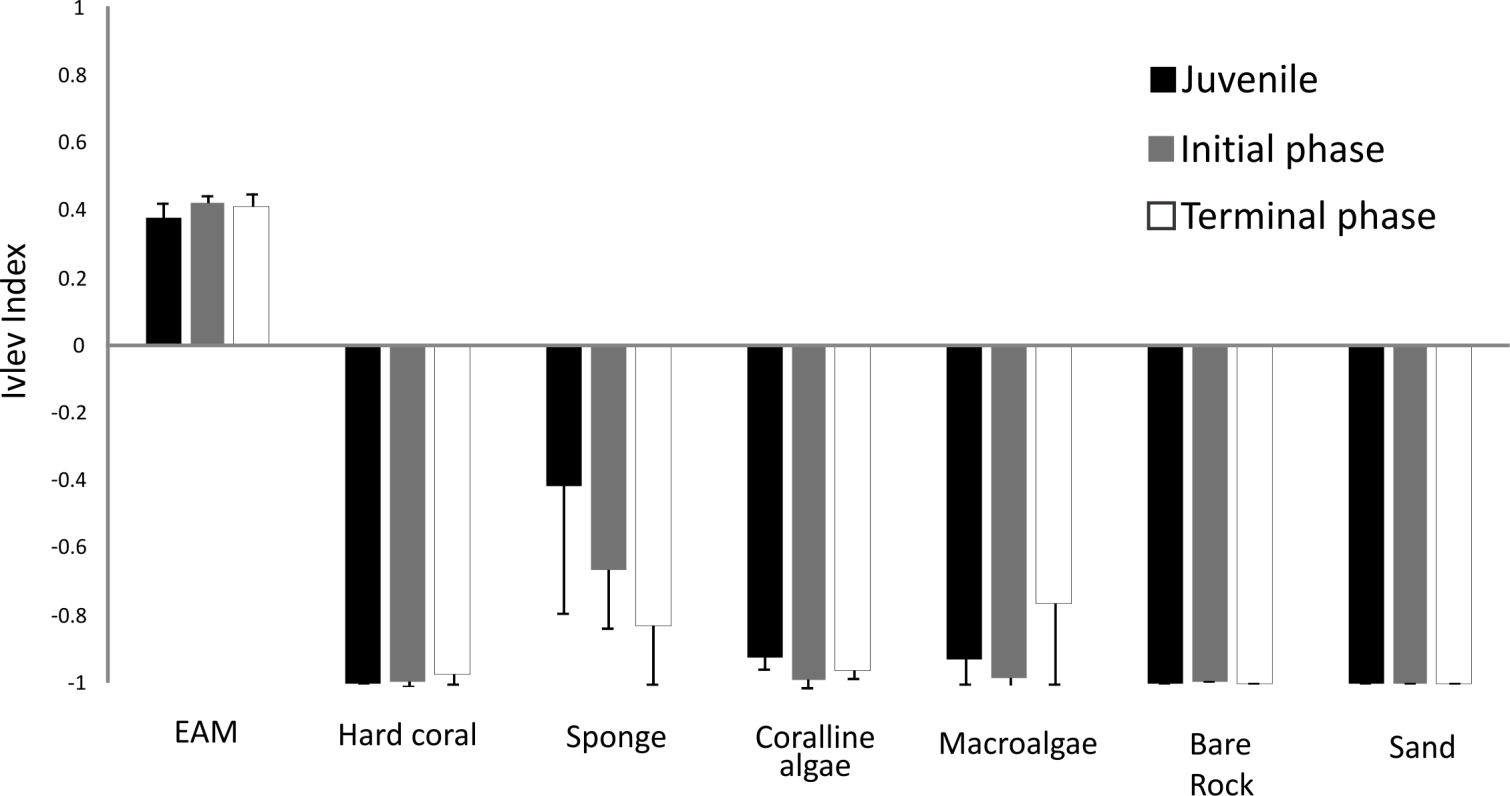
Ivlev’s electivity index of *S. zelindae* based off relative feeding rates and relative abundance of the benthos composition at foraging site. Bars in the figure represent 95% confidence intervals.

*S. zelindae* individuals at different life phases foraged selectivity on different benthos. Based on the Ivlev’s electivity index, juveniles selected EAM and sponge, whereas initial phase and terminal phase individuals selected only EAM (Fig. 5). The benthic categories sand, bare rock, coralline algae and hard coral were avoided completely by all life phases. Sponge and macroalgae were also avoided, yet to somewhat different degrees by different life stages (Fig. 5).

## Discussion

Parrotfish populations are under intense decline in the southwestern Atlantic Ocean, with many species suffering up to 50% reduction in total abundance in recent decades (Floeter et al., 2008; Bender et al., 2014). Despite this evident decline, baseline knowledge on parrotfish ecological role, such as foraging activity and ontogenetic changes in resource use are still scarce in the Atlantic Ocean outside the Caribbean. Our findings demonstrate that the feeding rate of *S. zelindae* decreases as body size increases. Additionally, EAM was the preferred foraging benthos for all life phases, with lower rejection of sponge in juveniles and macroalgae in terminal phase individuals. It is worth mentioning that Ivlev’s electivity index (i.e., foraging selectivity) accounts for resource/food availability, and thus characterizes true foraging preferences. Understanding variation in foraging can inform how fishing, which targets adult parrotfish, alter the ecological role of parrotfishes, which normally prevents macroalgae from displacing corals, thereby enhancing the resilience of coral reefs. Removal of large parrotfish due to fishing can cause a release of grazing pressure on EAM, thus allowing macroalgae to grow and outcompete corals.

Ecomorphological patterns of many southwestern Atlantic Ocean parrotfish species were recently analysed by Lellys (2014) using premaxilla, dentary and mouth configuration data. Lellys (2014) determined that the weaker and more mobile oral apparatus of smaller *S. zelindae* individuals classify them as *scrapers*. Additionally, the broad cutting edges of teeth in small *S. zelindae* individuals increases the contact area of the jaw, spreading the force over the substrate during feeding and therefore reducing bite force (Bellwood & Choat, 1990; Lellys, 2014). In contrast, according to Francini-Filho et al. (2010), *S. zelindae* terminal phase individuals could be classified as *excavators*, feeding at low rates and removing large portions of the substratum using their robust jaws, leaving noticeable scars. Our results confirm the findings of Francini-Filho et al. (2010) that the lowest feeding rates were observed for terminal phase individuals that foraged primarily on EAM and coralline algae. Larger parrotfish may feed at lower rates because they are able to acquire large amounts of food per bite, taking fewer yet larger bites.

Additionally, we have observed large *S. zelindae* individuals removing portions of the substratum, leaving feeding scars (e.g., *Siderastrea stellata* coral colonies). Although variations in *S. zelindae* bite size were not analysed in the present study, terminal phase individuals could have a greater effect on benthic communities compared to juveniles and initial phase individuals due to larger jaw size, as previously shown for other parrotfishes (Bonaldo, Hoey & Bellwood, 2014). Hence, larger bodied individuals are not only likely taking larger bites but those bites are likely having a larger impact on the benthos due to force/bite intensity. Future research using bite excavation measurements could elucidate this impact on benthic communities (e.g., bioerosion) and test the hypothesis that adults, normally targeted by local fisheries, could be the most effective individuals controlling algal growth.

Terminal phase individuals recorded in the present study displayed lower feeding rates compared to juveniles and initial phase individuals. This could be associated with patrolling behaviour observed for larger parrotfish size classes, on a few occasions during this study, which is likely to reduce their feeding rates once energy is allocated for mating and patrolling (Van Rooij, Kroon & Videler, 1996; Bonaldo et al., 2006). Haremic parrotfish also tend to increase their territory size and therefore more time should be used to protect this area (Mumby & Wabnitz, 2002). Additionally, it has been suggested recently that observer presence could reduce feeding rates of fishes on coral reefs (Pereira, Leal & Araújo, 2016). Consequently, the impact of observer presence could be intensified on terminal phase individuals who are normally patrolling much more often than individuals of other size classes.

Spatial variation in the availability of benthic resource could potentially influence *S. zelindae* feeding preference during the present study. Therefore, we have assessed the benthic community in foraging areas, to understand the ontogenetic selective patterns according to resource availability. According to Bonaldo, Hoey & Bellwood (2014) the availability and productivity of surfaces covered by EAM, the main feeding substratum for most parrotfish, may directly influence the distribution and feeding habitats of parrotfish. It is likely that EAM could be the most important food resource for the Brazilian endemic parrotfish throughout the lifespan of the specie due to their natural preference for and availability of EAM. However, juveniles also foraged at sponges. The use of sponges as a food resource for juvenile parrotfishes is uncommon; therefore, juveniles could be foraging on the mucus associated with the sponges as well as ingesting algae biofilm that grow on top of sponges (Randall & Hartman, 1968; Wulff, 2006). Similarly, Pereira et al. (2012) observed juveniles of *S. zelindae* feeding on *Millepora* spp. fire-corals on Brazilian coral reefs.

Fishing intensity on coral reefs (mainly spearfishing) normally targets larger, terminal phase parrotfish. According to Nunes et al. (2012) recreational spearfishing often captures endemic and larger herbivorous species in Brazilian waters, such as the endangered species *Scarus trispinosus* (Labridae). During many years of diving on the coral reefs analysed in the present study, only a few rare individuals of *Scarus trispinosus* were recorded. Additionally, following interviews conducted with the local community in 2015, a dramatic reduction in the abundance of this endangered species was reported (PHC Pereira, 2014, unpublished data). Hence, *Scarus trispinosus* is becoming functionally extinct in Pernambuco state outside of non-take zones, which is a troubling trajectory that *S. zelindae* population seems to also be following. The herbivore community at the deeper reefs (>25m) was previously analysed in a pilot study and the three most current abundant species were *Sparisoma axillare* (7.01 ind./100m^2^), *Scarus zelindae* (6.28 ind./100m^2^) and *Sparisoma frondosum* (3.39 ind./100m^2^) (PHC Pereira, 2015, unpublished data). By removing larger bodied individuals of parrotfish we could be losing a unique and critical functional group on southwestern Atlantic Ocean.

The creation of new marine protected areas ranks within priority actions for reef fish conservation in Brazilian waters, due to high levels of endemism (up to 30% in reef fishes) (Floeter et al., 2008; Schiavetti et al., 2013). However, effective management of the few existing marine protected areas in Brazil represent the most urgent conservation action to protect *S. zelindae* and other large Brazilian endemic parrotfish (Francini-Filho et al., 2010). Despite the fact that the reefs we studied are included in the largest Brazilian marine protected area (MPA), the abundance of large herbivores has been dramatically reduced in recent decades. This trend highlights the fact that the creation of more MPAs is probably not the most effective way to increase protection of endangered coral reef fishes. Accordingly, it is important to increase surveillance and monitoring of existing MPAs. Environmental education programmes and alternative livelihoods for local communities are also important strategies to reduce fishing pressure on endangered parrotfish species as previously observed in other developing countries such as Kenya (Cinner et al., 2012; Carter & Garaway, 2014), and Thailand (Bennett & Dearden, 2014).

Much discussion has arisen, mainly in the last decades, regarding the abundance of parrotfishes and the resilience of coral reef ecosystems. Nevertheless, Adam et al. (2015) suggested in a recent review that the evidence is mixed in showing that increases in herbivory can promote coral recovery on Caribbean reefs. The impacts of herbivores on coral reef ecosystems will vary greatly in space and time and will depend on herbivore diversity and species identity. Additionally, the findings of Suchley, McField & Alvarez-Filip (2016) contrast with the top-down herbivore control paradigm of coral reef and suggest that the role of external factors could be important in making environmental conditions more favourable for algal growth. Brazilian coral reefs are dominated by high abundance of macroalgae which seems to explain a large proportion of variance in reef fish abundance and species richness (Pereira et al., 2014). Therefore, it is critical to better understand the ecological role of parrotfishes and the ontogenetic influence of these species on algal dominated reefs throughout the southwestern Atlantic Ocean.

##  Supplemental Information

10.7717/peerj.2536/supp-1 Data S1 Raw data of *S. zelindae* foraging behaviour

10.7717/peerj.2536/supp-2 Data S2 Raw data of benthic cover

## Additional Information and Declarations

### Field Study Permissions

The following information was supplied relating to field study approvals (i.e., approving body and any reference numbers): BioBrazil - 24953. (SiSBio –16109).

### Data Availability

The following information was supplied regarding data availability: The raw data has been supplied as Supplemental Files and a video is available: Recifes Profundos - PCR 2016: https://www.youtube.com/watch?v=hDku5kmWVb4.
